# Mitochondria and oxidative stress in epilepsy: advances in antioxidant therapy

**DOI:** 10.3389/fphar.2024.1505867

**Published:** 2025-03-19

**Authors:** Delphine Ji, Shanthini Mylvaganam, Prathyusha Ravi Chander, Mark Tarnopolsky, Keiran Murphy, Peter Carlen

**Affiliations:** ^1^ Krembil Research Institute, Toronto, ON, Canada; ^2^ Departments of Medicine and Physiology, University of Toronto, Toronto, ON, Canada; ^3^ Department of Pediatrics, McMaster Children’s Hospital, Hamilton, ON, Canada; ^4^ Biomedical Engineering, University of Toronto, Toronto, ON, Canada

**Keywords:** epilepsy, antioxidant therapy, oxidative stress, mitochondrial dysfunction, reactive oxygen species

## Abstract

Epilepsy, affecting approximately 50 million individuals worldwide, is a neurological disorder characterized by recurrent seizures. Mitochondrial dysfunction and oxidative stress are critical factors in its pathophysiology, leading to neuronal hyperexcitability and cell death. Because of the multiple mitochondrial pathways that can be involved in epilepsy and mitochondrial dysfunction, it is optimal to treat epilepsy with multiple antioxidants in combination. Recent advancements highlight the potential of antioxidant therapy as a novel treatment strategy. This approach involves tailoring antioxidant interventions—such as melatonin, idebenone, and plant-derived compounds—based on individual mitochondrial health, including mitochondrial DNA mutations and haplogroups that influence oxidative stress susceptibility and treatment response. By combining antioxidants that target multiple pathways, reducing oxidative stress, modulating neurotransmitter systems, and attenuating neuroinflammation, synergistic effects can be achieved, enhancing therapeutic efficacy beyond that of a single antioxidant on its own. Future directions include conducting clinical trials to evaluate these combination therapies, and to translate preclinical successes into effective clinical interventions. Targeting oxidative stress and mitochondrial dysfunction through combination antioxidant therapy represents a promising adjunctive strategy to modify disease progression and improve outcomes for individuals living with epilepsy.

## Introduction

Epilepsy is a common and complex neurological disorder characterized by chronic, unprovoked seizures and affects approximately 50 million individuals worldwide (World Health, 2019). It is defined by the International League Against Epilepsy (ILAE) as transient occurrences of signs or symptoms due to abnormal excessive or synchronous neuronal activity in the brain (Scheffer et al., 2017). The hallmark characteristic is spontaneous recurrent seizures (SRS), corresponding with increased neuronal synchrony and excitability. The etiology ranges from genetic mutations, structural abnormalities, and, importantly, metabolic and/or mitochondrial dysfunction. Since the brain already has high aerobic metabolic demands and polyunsaturated fatty acids, it becomes particularly susceptible to insult and functional disturbances (Patel, 2002; Rho and Boison, 2022) (Supplementary Table S1; Table 1).

**TABLE 1:** Summary of clinical trials investigating antioxidant therapies in epilepsy.

NCT Number	Study title	Conditions	Antioxidant(s) used	Status	Key findings/Notes
NCT05987397	Exploring the Preventive Effect of Mitochondrial Protective Agent Idebenone on Post-stroke Epilepsy	Post-stroke Epilepsy	Idebenone	Recruiting	Ongoing study; results not yet available. Idebenone is investigated for its neuroprotective antioxidant properties
NCT05654415	Melatonin vs. Sleep Deprivation for Nap EEG	Epilepsy	Melatonin	Active, not recruiting	Comparing the effectiveness of melatonin versus sleep deprivation in inducing sleep for EEG recordings in epilepsy patients. Results pending
NCT05637086	Clinical Study Evaluating Safety of Pentoxifylline and Celecoxib in Patients With Grand-Mal Epilepsy Treated by Phenytoin Monotherapy	Epilepsy	Pentoxifylline, Celecoxib	Recruiting	Evaluating safety of anti-inflammatory agents pentoxifylline and celecoxib as adjunct therapy. Results not yet available
NCT05485558	The Safety and Efficacy of N-Acetyl Cysteine in Children With Drug-Resistant Epilepsy	Drug-Resistant Epilepsy	N-Acetyl Cysteine (NAC)	Recruiting	Assessing NAC’s antioxidant effects on seizure control in children with drug-resistant epilepsy. Results pending
NCT04665453	Dexmedetomidine and Melatonin for Sleep Induction for EEG in Children	Epilepsy, EEG Sleep Induction	Melatonin	Completed	Studied melatonin’s efficacy in inducing sleep for EEG procedures in pediatric epilepsy patients. Results may provide insights into melatonin’s sedative properties
NCT04545346	The Potential of a Low Glutamate Diet as a Treatment for Pediatric Epilepsy	Pediatric Epilepsy	Low Glutamate Diet	Completed	Investigated dietary intervention to reduce excitatory neurotransmission. Findings may inform nutritional approaches in epilepsy management
NCT03776656	Evaluation of a Treatment With Allopurinol in Adenylosuccinate Lyase Deficiency	Adenylosuccinate Lyase Deficiency	Allopurinol	Completed	Explored allopurinol’s potential to reduce oxidative stress in a metabolic disorder associated with epilepsy. Results could have implications for antioxidant therapy
NCT03590197	Effect of Melatonin on Seizure Outcome, Neuronal Damage, and Quality of Life in Patients With Generalized Epilepsy	Generalized Epilepsy	Melatonin	Completed	Evaluated melatonin’s impact on seizures, neuronal protection, and patient quality of life. Awaiting published results
NCT01764516	Study on Serum Zinc and Selenium Levels in Epileptic Patients	Generalized Epilepsy	Zinc, Selenium	Completed	Investigated antioxidant mineral levels in patients with epilepsy. Findings may highlight the role of micronutrient status in epilepsy
NCT01161108	Trial of Melatonin to Improve Sleep in Children With Epilepsy and Neurodevelopmental Disabilities	Epilepsy, Developmental Disability, Insomnia	Melatonin	Completed	Assessed melatonin’s effectiveness in improving sleep quality in children with epilepsy. Results could inform sleep management strategies
NCT00965575	Pilot Study of Melatonin and Epilepsy	Epilepsy	Melatonin	Completed (with results)	Results available; investigated melatonin’s impact on seizure frequency and sleep patterns in epilepsy patients
NCT00678834	Human Tissue Distribution of Orally Supplemented Natural Vitamin E Tocotrienol	Various Conditions, Including Healthy Subjects	Vitamin E Tocotrienol	Completed (with results)	Studied distribution of vitamin E tocotrienol; may have implications for antioxidant therapy in neurological conditions
NCT00004637	Double-Blind, Placebo-Controlled Trial of Vitamin E as Add-on Therapy for Children With Epilepsy	Epilepsy	Vitamin E	Completed	Evaluated the efficacy of vitamin E as an adjunctive treatment in pediatric epilepsy. Results may indicate antioxidant benefits

Overview of clinical trials registered on ClinicalTrials.gov evaluating the efficacy and safety of various antioxidant therapies in individuals with epilepsy.

Although the exact mechanisms of epileptogenesis have not been fully elucidated, there is increasing evidence for the involvement of mitochondria as crucial organelles in cellular energy production. In neurons, mitochondria are essential for maintaining membrane potential, calcium regulation (Bierhansl et al., 2024), and bioenergetics that support the high energetic demands for synaptic transmission. Dysfunction of mitochondrial processes, such as in ATP generation, calcium buffering, and the regulation of apoptosis, are increasingly recognized as contributors to the development and progression of epilepsy, particularly in mitochondrial epilepsies (Lopriore et al., 2022). In primary mitochondrial diseases such as MELAS or MERRF syndromes, defects in oxidative phosphorylation (OXPHOS) can lead to ATP depletion, impairing neuronal hyperpolarization and contributing to excessive excitatory activity. This disruption of energy homeostasis not only changes sodium-potassium pump (Na^+^/K^+^ ATPase) activity but also leads to the death of inhibitory interneurons, which are particularly vulnerable to OXPHOS deficiencies (Reinecke et al., 2009). This results in reduced GABAergic inhibition, increased glutamate release from astrocytes, and an overall hyperexcitable network, lowering the seizure threshold.

## Oxidative stress and epilepsy

Oxidative stress plays a complex role in the pathophysiology of epilepsy, particularly in patients with mitochondrial dysfunction (Patel, 2002). Under normal physiological conditions, reactive oxygen species (ROS) and reactive nitrogen species (RNS) are produced as byproducts of cellular metabolism, especially in the mitochondrial respiratory chain, which accounts for many of the free radicals produced in the body (Juan et al., 2021; Kowalczyk et al., 2021). These species, including superoxide (O_2_^−^⋅), hydrogen peroxide (H_2_O_2_), and the hydroxyl radical (HO⋅), are involved in cellular signalling but can become harmful when produced in excess (Pizzino et al., 2017).

Specifically in mitochondria, superoxide radicals are generated through the reduction of molecular oxygen during electron transport (Turrens, 2003). While hydrogen peroxide is a by-product of this reaction and is not a free radical, it can be converted into hydroxyl radicals, some of the most damaging forms of ROS. The reaction between superoxide and nitric oxide (NO) forms peroxynitrite (ONOO^−^), a highly reactive molecule capable of initiating lipid peroxidation, protein nitration, and DNA damage (Castro and Freeman, 2001; Rowley et al., 2015). NADPH oxidase (Nox2) and cyclooxygenase-2 (COX-2) are additional enzymes that contribute to the oxidative stress burden in epilepsy (Rawat et al., 2019; Almeida et al., 2022). COX-2 is expressed in astrocytes, which are responsible for releasing proinflammatory cytokines, further exacerbating neuronal damage (Rawat et al., 2019). Oxidative stress in epilepsy results from an imbalance between pro-oxidant species and the potential of antioxidant defences that normally neutralize them. When this balance is disturbed, ROS and RNS can cause oxidative damage to DNA (Di Meo et al., 2016), proteins, and lipids, leading to cellular damage and apoptosis (Schieber and Chandel, 2014). Oxidative stress-induced damage has been implicated in numerous diseases, including epilepsy, atherosclerosis (Batty et al., 2022), diabetes complications (Volpe et al., 2018), and cancer (Glorieux et al., 2024). In the literature, the role of free radicals in causing malondialdehyde (MDA) elevation is particularly significant in epilepsy, as MDA is a marker of lipid peroxidation and higher levels have been observed in patients with recurrent seizures (Yilgor and Demir, 2024).

More specifically in epilepsy, oxidative stress can further impair mitochondrial function and set up a “vicious cycle”, leading to metabolic disturbances in neurons. It is generally understood that low levels of antioxidant enzymes such as superoxide dismutase (SOD) and catalase (CAT), combined with high levels of MDA, are indicative that the antioxidant defence system is overwhelmed by ROS production (Yilgor and Demir, 2024). Non-monogenic epilepsies are well-documented to be associated with mitochondrial dysfunction. There is evidence from both preclinical (Supplementary Table S1) and clinical studies (Table 1) that oxidative stress plays a key role in the initiation and progression of epilepsy. This is primarily founded on animal models using kainic acid or pilocarpine models of status epilepticus (Dal-Pizzol et al., 2000).

Mitochondrial disorders are frequently associated with epilepsy, and, conversely, seizures are associated with causing mitochondrial dysfunction with oxidative stress (Kunz, 2002; Patel, 2002). It has been hypothesized that impaired mitochondrial energy production could be the basis of pharmacoresistance in epilepsy (Yuen and Sander, 2011). The standard method of treating epilepsy patients consists of successive trials of many antiepileptic drugs, most of which raise the seizure threshold without addressing other aspects of their disorder, such as pervasive mitochondrial dysfunction. Status epilepticus (SE), sometimes fatal, is associated with oxidative stress, bioenergetic failure, and impaired mitochondrial dynamics in both mature and immature brains, all ameliorated by antioxidant treatments (Folbergrová and Kunz, 2012). Remarkably, antioxidant therapy is not used in SE, a treatment which could be quite safe and easily translatable. As argued below, it is becoming clear that the approach of using multiple rather than one or two antioxidants appears to be an improved approach for treating the many facets of mitochondrial dysfunction in epilepsy, as well as other neurological/neurodegenerative disorders. Neuroprotectants, particularly antioxidants, may have therapeutic value in epilepsy by reducing oxidative stress and its damaging effects on neurons. Increased levels of antioxidant enzymes such as SOD, CAT, and glutathione (GSH) have been proposed as potential therapeutic targets in refractory epilepsy (Cardenas-Rodriguez et al., 2013).

## Rationale for personalized antioxidant therapy

Predictive, preventive, and personalized medicine (PPPM/3PM) is a shift from the traditional “one-size-fits-all” approach in diseases that involve significant metabolic dysfunctions like mitochondrial diseases (MDs). Mitochondrial genetics significantly affect individual responses to therapies, including antioxidant treatments, which are increasingly being explored for their potential to counteract mitochondrial oxidative stress. One example includes mitochondria being a critical target in the context of hypoxic and ischemic injury, being central to therapeutic strategies aimed at improving outcomes in stroke patients (Ham III and Raju, 2017). Mitochondrial health and quality control are pivotal not only for assessing the risk of ischemic stroke but also for protecting neural tissue, supporting survival, and enhancing recovery outcomes on a personalized basis (Anzell et al., 2018; He et al., 2020). Furthermore, damage to the blood-brain barrier in the peri-infarct area, which often results in secondary injury, is strongly associated with limited recovery and significant disruptions in mitochondrial function (Nahirney et al., 2016). Another example is shown in tuberculosis (TB) and the body’s redox response to infection. The non-protein thiol glutathione (GSH) protects against *Mycobacterium tuberculosis* (MTB) infection. GSH, in conjunction with the transcription factor Nrf2 (nuclear factor erythroid 2-related factor 2), are crucial in counteracting the redox imbalance induced by MTB. Nrf2 mediates the expression of numerous antioxidant genes, and its antioxidant response element (ARE) signalling pathway is increasingly recognized as central to the pathogenesis of TB (Palanisamy et al., 2011). Personalized modulation of Nrf2-target genes highlights the potential of antioxidant therapies to enhance the efficacy of TB treatments (Petrillo et al., 2022).

## Mitochondrial variability in epilepsy

Mitochondrial DNA (mtDNA) mutations have been increasingly associated with many neurological disorders, including epilepsy. While most genetic neurological conditions are linked to nuclear DNA mutations, defects in mtDNA significantly contribute to diseases such as Autism Spectrum Disorder (ASD) (Varga et al., 2018; Wang et al., 2022), Huntington’s disease (Ayala-Peña, 2013; Neueder et al., 2024), bipolar disorder (Munakata et al., 2004), and Leigh Syndrome (Ball et al., 1993). The mtDNA is a circular, double-stranded molecule comprising 16,659 nucleotides. It encodes 13 protein-coding genes essential for the electron transport chain (ETC.) complexes I, III, IV, and V, crucial for OXPHOS and ATP production (Anderson et al., 1981). Mutations in these genes can impair mitochondrial function, leading to decreased ATP production and increased ROS generation, contributing to neuronal hyperexcitability and seizures (Larsson et al., 1998). Specific mtDNA mutations are linked to mitochondrial encephalomyopathies associated with epilepsy, notably Mitochondrial Encephalopathy, Lactic Acidosis, Stroke-like episodes (MELAS) and Myoclonic Epilepsy with Ragged Red Fibers (MERRF) syndromes (Zeviani et al., 1993). In MELAS, the m.3243A > G mutation in the tRNA^Leu(UUR) gene affects mitochondrial protein synthesis, leading to defective OXPHOS and increased oxidative stress (Pia and Lui, 2024). MERRF is commonly associated with the m.8344A > G mutation in the tRNA^Lys gene, resulting in similar mitochondrial dysfunction (Hameed and Tadi, 2024). Variations in mtDNA can also influence mitochondrial biogenesis, apoptosis, calcium ion regulation, and other essential cellular processes (Osellame et al., 2012). Heteroplasmy, the coexistence of mutant and wild-type mtDNA within cells, contributes to variability in clinical presentations and disease severity (Stewart and Chinnery, 2015). In rat models, heteroplasmy has been shown to alter metabolic function, causing behavioural and cognitive deficits (Stewart and Chinnery, 2015).

Mitochondrial haplogroups, defined by specific mtDNA polymorphisms inherited maternally, may influence susceptibility to oxidative stress and response to antioxidant therapies (Gómez-Durán et al., 2010). Different haplogroups can affect mitochondrial efficiency and ROS production, potentially altering an individual’s vulnerability to mitochondrial dysfunction-related epilepsy (Amo et al., 2008). Certain haplogroups may be associated with higher baseline ROS production due to less efficient electron transport, increasing oxidative stress and seizure susceptibility; however, in cases of neurodegeneration, the potential as a risk factor remains controversial (Mancuso et al., 2008; Ingram et al., 2012). Understanding the role of mitochondrial haplogroups in epilepsy could aid in predicting disease risk and tailoring antioxidant therapies (Rea et al., 2013). Mitochondrial dysfunction resulting from genetic mutations leads to increased ROS production, contributing to neuronal damage and epileptogenesis (Soini et al., 2013). Antioxidant therapies have been explored to mitigate oxidative stress in epilepsy, but responses vary based on mitochondrial genetic background (Vergani et al., 2004). Studies have demonstrated that patients with mitochondrial disorders respond differently to antioxidants like Coenzyme Q10 (CoQ10) (Quinzii and Hirano, 2010). In cases of primary CoQ10 deficiency, resulting from mutations in nuclear genes involved in CoQ10 biosynthesis (e.g., *PDSS2*, *COQ9*, *ADCK3*), supplementation with CoQ10 has led to improvements in animal experiments, including reduced seizure duration (Sattarinezhad et al., 2014; Simani et al., 2020) and has been measured in various mitochondrial disorders in an RCT (Glover et al., 2010). In mitochondrial disorders caused by mtDNA mutations, such as MELAS, the efficacy of antioxidant therapies shows some evidence (Rodriguez et al., 2007). The variable responses may be due to differences in how specific mutations affect mitochondrial function and the resulting oxidative stress levels. Given the heterogeneity of mitochondrial genetic defects, personalized antioxidant therapies hold promise for improving treatment outcomes. By identifying specific mitochondrial mutations or haplogroups present in a patient, clinicians can tailor antioxidant strategies to target the underlying mitochondrial dysfunction more effectively (Meng et al., 2021). For instance, patients with mutations leading to deficiencies in, ETC complexes might benefit from antioxidants that support electron transport and reduce ROS production. Mitochondrial-targeted antioxidants, such as MitoQ or SkQ1, are designed to accumulate within mitochondria and directly neutralize ROS at the source (Murphy and Smith, 2007; Skulachev et al., 2023). These targeted therapies could enhance treatment efficacy and reduce potential toxicity associated with higher doses of non-specific antioxidants (Wang et al., 2011).

## Synergistic benefits in treating epilepsy

The potential for the use of multi-ingredient supplements to target the multiple final common pathways of neuronal dysfunction was first proposed in 2001 by Tarnopolsky and Beal (2001). Others have supported this contention, suggesting that synergism occurs when the combined effect of multiple antioxidants is greater than the sum of their individual effects (Thoo et al., 2013). This phenomenon can arise from various mechanisms, including antioxidant regeneration, differences in cellular localization, and complementary actions on oxidative pathways (Wang et al., 2011). For instance, one antioxidant may regenerate another by donating electrons to restore its active form, thereby extending its antioxidant activity. Several mechanisms contribute to this improved synergism such as redox cycling, antioxidant partitioning, and various formulations and combinations. In redox cycling, one antioxidant regenerates another by donating electrons, restoring its active form. For example, ascorbic acid (vitamin C) can regenerate α-tocopherol (vitamin E) by reducing the α-tocopheroxyl radical back to α-tocopherol (Niki, 1987). This process maintains antioxidant activity and prolongs protection against oxidative damage. Antioxidants with varying solubilities will also localize differently within biological systems, targeting oxidative stress in multiple compartments. Lipid-soluble antioxidants protect cell membranes, while water-soluble antioxidants defend the cytosol (Sharifi-Rad et al., 2020). Their distinct localization can enhance overall antioxidant efficacy. Together, antioxidants that scavenge free radicals and those that chelate pro-oxidant metal ions can more effectively reduce oxidative stress by addressing multiple pathways simultaneously (Halliwell, 1987). The concept of the use of multi-ingredient supplements for genetic mitochondrial disease was first studied in a randomized, double-blind study showing that the use of a multi-ingredient supplement that provided an alternative energy source (creatine monohydrate) + a membrane anti-oxidant (vitamin E) and two mitochondrial localized anti-oxidants/redox couple (COQ10 + alpha lipoic acid) lowered ROS markers and lactate (improved mitochondrial function) (Rodriguez et al., 2007; Tarnopolsky and Beal, 2001). Support for the superiority of the multi-ingredient supplement approach vs. a single agent targeting one pathway (ROS) was reflected in the fact that very high doses of CoQ10 (600 mg bid) neither lowered oxidative stress nor lactate in a similar cohort genetic mitochondrial disease patients (Glover et al., 2010).

For example, the combination of ascorbic acid and α-tocopherol has shown synergistic antioxidant effects in protecting phospholipid bilayers (Liebler et al., 1986). Ascorbic acid regenerates α-tocopherol from its radical form, sustaining membrane protection against lipid peroxidation. Flavonoids like quercetin and myricetin, which have lower redox potentials than α-tocopherol, can regenerate α-tocopherol and enhance its antioxidant activity (Marinova et al., 2008). Studies have reported synergistic interactions between α-tocopherol and flavonoids in inhibiting lipid oxidation (Bayram and Decker, 2023). Combining mitochondrial-targeted antioxidants with agents that modulate gene expression can restore mitochondrial function more effectively. Activation of Nrf2, a transcription factor that upregulates antioxidant defences, has shown promise in enhancing cellular resilience to oxidative stress (Li and Kong, 2009). Epigenetic therapies influence gene expression without altering the DNA sequence, affecting pathways involved in oxidative stress and mitochondrial health (Shaughnessy Daniel et al., 2014). By combining antioxidants with epigenetic modulators, it is possible to target genetic pathways that restore mitochondrial function and reduce seizure susceptibility. Nrf2 controls the expression of antioxidant enzymes and cytoprotective proteins by activating an ARE (Cardenas-Rodriguez et al., 2013). Activating Nrf2 enhances the cell’s endogenous antioxidant capacity. Compounds like RTA 408, an Nrf2 activator, have shown neuroprotective effects in preclinical models (Shekh-Ahmad et al., 2019). NOX enzymes are significant sources of ROS in the central nervous system. Inhibiting NOX reduces ROS generation during seizures. Agents like AEBSF, a NOX inhibitor, can decrease oxidative damage when combined with antioxidants (Shekh-Ahmad et al., 2019). This combination prevented seizure-induced mitochondrial depolarization, ROS generation, and neuronal cell death more effectively than either agent alone. *In vivo*, the combination therapy increased antioxidant capacity following kainic acid (KA)-induced SE, prevented the development of epilepsy, and reduced seizure frequency in established epilepsy models (Shekh-Ahmad et al., 2019).

The use of multiple antioxidants offers a promising strategy to address the complex pathophysiology of epilepsy, particularly in cases where personalized medicine is not feasible. Mitochondrial dysfunction plays a central role in epilepsy, even in monogenic forms of the disease, with downstream effects including excitotoxicity, calcium dysregulation, excessive reactive oxygen species (ROS) production, and neuroinflammation. Combining antioxidants that target diverse pathways associated with these dysfunctions can enhance therapeutic efficacy. Evidence from animal models and clinical studies highlights the potential of antioxidants such as vitamin E, melatonin, coenzyme Q10, and polyphenols to reduce seizure frequency and severity. However, when used as monotherapies, antioxidants may exhibit pro-oxidant effects under certain conditions, as demonstrated by Tarnopolsky (Tarnopolsky, 2008), making combination therapies a safer and more effective approach. Like “mitochondrial cocktails,” multi-antioxidant regimens provide broad-spectrum coverage, mitigating oxidative stress while modulating neuroinflammatory and neurotransmitter pathways. This strategy has shown promise in epilepsy-related dietary interventions like the ketogenic diet, which enhances mitochondrial health (Miller et al., 2020). Additionally, a multi-antioxidant approach can address comorbidities frequently associated with mitochondrial dysfunction, such as cognitive decline and mood disorders (Fattal et al., 2007). Further, by combining multiple antioxidants to target various regions in the oxidative and inflammatory cascades, antioxidant therapies can synergistically counteract the multifactorial nature of epilepsy pathogenesis, offering a robust alternative when personalized treatments are unattainable. This has been introduced as a preventative method from DNA injury in diagnostic radiation exposure (Merlin et al., 2022; Xhuti et al., 2023).

## Preclinical models of antioxidant use

A substantial body of preclinical research has explored the therapeutic potential of antioxidant compounds in managing epilepsy. These studies have employed animal models to investigate how antioxidants can potentially mitigate seizure activity, prevent neuronal damage, and modulate oxidative stress and neuroinflammatory pathways associated with epileptogenesis.

Natural antioxidants derived from plants and other sources have been featured in many of these articles. For instance, royal jelly (RJ) demonstrated significant neuroprotective effects in kainic acid-induced TLE in rats by reducing seizure severity and oxidative stress markers, while enhancing total antioxidant capacity and preventing hippocampal neuronal damage (Hashemi et al., 2023). Proanthocyanidins (PACs) exhibited anticonvulsant effects in pentylenetetrazole (PTZ)-induced epilepsy in mice through activation of the Nrf2 pathway, leading to decreased oxidative stress, inflammation, and neuronal apoptosis (Alyami et al., 2022). Sulforaphane (SFN), another Nrf2 activator, reduced ROS production, restored glutathione levels, and attenuated neuronal death in kainic acid-induced SE in rats (Sandouka and Shekh-Ahmad, 2021). Other plant-derived compounds, such as curcumin derivatives, lycopene, and extracts from *Melissa officinalis*, *Echinops spinosus*, and *Syzygium cumini*, have also shown significant anticonvulsant and neuroprotective effects. These effects are primarily mediated through antioxidant mechanisms, modulation of neurotransmitter systems (e.g., GABA), and attenuation of neuroinflammation (Mahmoudi et al., 2020; Taskiran and Tastemur, 2021; Abd Allah et al., 2022; Kandeda et al., 2022; Alkhudhayri et al., 2023). Synthetic antioxidants and pharmaceuticals have been evaluated for their efficacy in epilepsy models as well. Tempol, a membrane-permeable radical scavenger, could attenuate PTZ-induced seizures in mice by reducing oxidative and nitrosative stress, enhancing GABAergic neurotransmission, and inhibiting pro-inflammatory cytokines (Zhang et al., 2018). Lacosamide, an antiepileptic drug (AED), not only decreased seizure activity in pilocarpine-induced SE in rats but also exerted antioxidant effects by restoring superoxide dismutase (SOD) activity and glutathione (GSH) levels (Shishmanova-Doseva et al., 2021). Mitochondrial dysfunction has emerged as a critical factor in epileptogenesis. Succinate accumulation contributed to increased oxidative stress and mitochondrial ROS levels, leading to neuronal degeneration and SE in kainic acid-induced models. Inhibiting succinate dehydrogenase (SDH) and related metabolic pathways reduced seizure severity and oxidative damage (Zhang et al., 2020). Interventions targeting mitochondrial bioenergetics, such as treatment with ascorbic acid, alpha-tocopherol, and sodium pyruvate (AATP), improved mitochondrial function, reduced seizure burden, and enhanced synaptic activity in temporal lobe epilepsy models (Simeone et al., 2014). Combination antioxidant therapies targeting multiple pathways have shown promise in providing enhanced neuroprotection. Preservation of ion channel function and enzyme activities has also been a focus. Agents like lipoic acid (LA) and idebenone prevented seizures and restored the activities of critical enzymes such as Na⁺/K⁺-ATPase and δ-aminolevulinic acid dehydratase (δ-ALA-D), which are essential for maintaining neuronal excitability and metabolic homeostasis (de Sales Santos et al., 2010; Ahmed, 2014).

## Future directions

Despite the promising results from preclinical studies demonstrating the neuroprotective and anticonvulsant effects of antioxidant therapies in epilepsy, several challenges hinder the translation of these findings into clinical practice. One significant challenge lies in the selective uptake limitations of mitochondria-targeted antioxidants. Damaged mitochondria, which typically exhibit lower membrane potential, may uptake these antioxidants less efficiently than their healthy counterparts, thereby reducing the efficacy of treatments aimed at mitigating oxidative stress within the very mitochondria that require intervention (Plotnikov and Zorov, 2019). Additionally, there is a risk of reductive stress, where excessive antioxidant supplementation disrupts the delicate balance of reactive oxygen species (ROS) necessary for normal cellular signaling and physiological functions. Over-suppression of ROS can impair essential processes such as cell differentiation, apoptosis, and immune responses, potentially leading to adverse cellular outcomes. There is also a potential for prooxidant activity under certain conditions, such as high concentrations or the presence of transition metals, which can paradoxically exacerbate oxidative stress rather than mitigate it (Podmore et al., 1998).

Determining the optimal dosage and administration regimen is complex, as factors such as bioavailability, pharmacokinetics, and individual patient variability influence therapeutic outcomes. Additionally, the lack of standardized methods for evaluating the efficacy and safety of these antioxidants poses significant regulatory challenges, making it difficult to establish universally accepted guidelines for their use. Addressing these disadvantages requires a multifaceted approach. Future research should focus on conducting clinical trials to evaluate the efficacy and safety of antioxidant compounds in patients with epilepsy. Personalized medicine holds great potential in optimizing antioxidant therapies, considering the variability in mitochondrial genetics among individuals. Exploring the role of mitochondrial DNA mutations and haplogroups in influencing the response to antioxidant treatments could enable the tailoring of therapies to individual patient profiles. Identifying oxidative stress and mitochondrial dysfunction biomarkers may further aid in customizing antioxidant interventions, enhancing therapeutic outcomes.

Moreover, combining antioxidants with anti-inflammatory agents or traditional antiepileptic drugs may provide synergistic effects, as suggested by preclinical studies (Pauletti et al., 2019; Shekh-Ahmad et al., 2019). The development of novel mitochondria-targeted antioxidants, such as MitoQ and SkQ1, offers the potential for a more effective reduction of oxidative stress at its primary source within neurons (Snow et al., 2010). Additional research is needed to understand the precise mechanisms by which antioxidants exert their anticonvulsant effects. Investigations into the role of the Nrf2 pathway, mitochondrial bioenergetics, and ion channel modulation in the context of antioxidant treatment could provide deeper insights (Waldbaum and Patel, 2010). Furthermore, the advantage of a multi-ingredient approach is notable, as it is likely to address a broader range of disorders compared to single-agent therapies. Given that mitochondrial genetic disorders, epilepsy, and most other neurological disorders converge on common pathways such as mitochondrial dysfunction, excitotoxicity, apoptosis, calcium dysregulation, ROS excess, and inflammation (Madireddy and Madireddy, 2023).

## Conclusion

Oxidative stress and mitochondrial dysfunction play critical roles in the pathophysiology of epilepsy, contributing to neuronal hyperexcitability and cell death. Preclinical studies have provided substantial evidence that antioxidant therapies can mitigate these pathological processes, reduce seizure activity, and protect neuronal integrity. Compounds such as melatonin, sulforaphane, and various plant extracts have demonstrated significant anticonvulsant and neuroprotective effects in animal models. While clinical trials investigating antioxidant therapies in epilepsy are limited, preliminary findings suggest potential benefits. However, more extensive clinical research is necessary to confirm these effects and to establish optimal dosing regimens, safety profiles, and patient selection criteria. Considering individual genetic and metabolic differences may enhance the efficacy of antioxidant treatments. Antioxidant therapies represent a promising adjunctive strategy in the management of epilepsy. By targeting oxidative stress and mitochondrial dysfunction, these agents have the potential to modify disease progression and improve patient outcomes. Continued research efforts are essential to translate preclinical successes into effective clinical interventions for individuals living with epilepsy.
