# Supplementary material for: Mitochondria and oxidative stress in epilepsy: advances in antioxidant therapy
Source: Front Pharmacol. 2025 Mar 19;15:1505867. doi: 10.3389/fphar.2024.1505867 (PMC11961640; doi:10.3389/fphar.2024.1505867)
Supplement: Supplementary file 1 [file Table1.docx]

Supplementary Table 1: Overview of Antioxidant Interventions in Animal Models of Epilepsy

| **Model/System** | **Antioxidant(s) Used** | **Reference** | **Key Findings** | **Proposed Mechanism** | **Dosage** |
| --- | --- | --- | --- | --- | --- |
| 6 Hz model of pharmacoresistant epilepsy in mice | 1,2,4-Triazole-3-thione derivatives | (Kaproń et al., 2020) | Compounds exhibited potent anticonvulsant activity, antioxidant/ROS scavenging activity, did not impair cognitive processes mediated by CAs, minimal off-target cholinergic effects. | Antioxidant properties contributing to anticonvulsant effects without cognitive side effects. | ED50  TP-10: Peak activity: 61.1 mg/kg (30 minutes after administration).  TP-315: Peak activity: 59.7 mg/kg (30 minutes after administration).  TP-427: Peak activity: 40.9 mg/kg (15 minutes after administration) |
| Kainic acid-induced seizures in rats | 2,2'-Dithienyl diselenide (DTDS) | (Bortolatto et al., 2011) | DTDS increased latency to seizures, decreased seizure severity, reduced EEG changes, ameliorated oxidative stress markers, maintained Na⁺/K⁺-ATPase activity, decreased hippocampal neurodegeneration. | Antioxidant effects; protection against oxidative stress and maintenance of Na⁺/K⁺-ATPase activity. | DTDS: 50 mg/kg (oral, p.o.), 100 mg/kg (oral, p.o.) |
| PTZ-induced seizures in mice | 4-Hydroxy-TEMPO (Tempol) | (Zhang et al., 2018) | Tempol (100 and 200 mg/kg) delayed onset of tonic-clonic convulsions, decreased duration and mortality. Reduced oxido-nitrosative stress, increased GABA and dopamine levels, increased Na⁺/K⁺-ATPase activity, decreased xanthine oxidase activity, downregulated pro-inflammatory cytokines and c-Fos expression. | Antioxidant, anti-inflammatory, and GABAergic effects contributing to anticonvulsant activity. | Tempol: 50 mg/kg (intraperitoneal, i.p.)  100 mg/kg (intraperitoneal, i.p.)  200 mg/kg (intraperitoneal, i.p.) |
| Temporal lobe epilepsy (TLE) in KO mice | AATP (ascorbic acid, alpha-tocopherol, sodium pyruvate) | (Simeone et al., 2014b) | AATP improved mitochondrial functions, reduced seizure burden and incidence, improved synaptic functions. | Restoration of bioenergetic homeostasis targeting mitochondrial impairments, reducing seizures. | Ascorbic Acid (Vitamin C): 250 mg/kg, intraperitoneally (i.p.)  Sodium Pyruvate: 500 mg/kg, i.p.  Alpha-Tocopherol (Vitamin E): 30 mg/kg, i.p. Chow: Enriched with ascorbic acid (2.5 g/kg) and alpha-tocopherol (0.30 g/kg).  Water: Enriched with sodium pyruvate (6.67 mg/mL) |
| PTZ-induced acute seizures in mice | Acetaminophen (pre-treatment) | (Karabulut and Taskiran, 2021) | Acetaminophen suppressed seizures, increased TAS and GABA levels, decreased TOS, NO, TNF-α, caspase-3, and glutamate levels in hippocampus. | Antiseizure potential through antioxidant, anti-inflammatory, and anti-apoptotic pathways. | 50 mg/kg AAP administered intraperitoneally (i.p.) for 5 consecutive days.  100 mg/kg AAP administered intraperitoneally (i.p.) for 5 consecutive days |
| PTZ-induced seizures in mice | Active fractions from *Anthocleista djalonensis* root bark | (Taiwe et al., 2021) | Fractions protected mice against seizures, decreased oxidative stress, increased GABA levels, reduced neuronal cell loss, and improved seizure parameters. | Enhancement of antioxidant defense and GABAergic signaling contributing to neuroprotection. | 20 mg/kg to 160 mg/kg, administered orally |
| PTZ-induced seizures in mice | *Albizia adianthifolia* aqueous extract | (Nkwingwa et al., 2023) | Extract protected mice against seizures, improved memory, reversed oxidative/nitrosative stress, GABA depletion, neuroinflammation, and neuronal cell death. | Anticonvulsant and anti-amnesic effects through amelioration of oxidative stress, GABAergic transmission, and neuroinflammation. | 40, 80, or 160 mg/kg |
| Kainic acid-induced TLE in rats | Alpha-pinene | (Hashemi and Ahmadi, 2023) | Alpha-pinene alleviated seizures, diminished oxidative stress indicators, blocked mitochondrial apoptotic pathway, decreased neuronal death. | Antioxidant effects and inhibition of JNK pathway reducing neuronal damage. | 50 mg/kg/day. Administered intraperitoneally for 2 weeks |
| PTZ-kindled epilepsy in mice | Apocynin (NADPH oxidase inhibitor) | (Jaiswal and Kumar, 2022) | Apocynin reduced seizure severity, oxidative stress, neurotransmitter alterations, comorbid conditions by inhibiting NADPH oxidase. | Neuroprotection through inhibition of ROS production via NADPH oxidase blockade. | 25, 50, and 100 mg/kg/day via intraperitoneal injection. The treatment was given daily for the duration of 1st to 33^rd^ day |
| Picrotoxin, PTZ, strychnine, and NMDA-induced seizures in mice; kainic acid-induced TLE | Aqueous and ethanol extracts of Nymphaea lotus | (Ishola et al., 2022) | Extracts prolonged seizure onset, reduced seizure duration, ameliorated oxidative stress, improved behavioral deficits, neurodegeneration reduced. Effects reversed by flumazenil or L-arginine. | Enhancement of GABAergic signaling, antioxidant effects, inhibition of glutamatergic neurotransmission. | 50, 100, and 200 mg/kg (p.o.) were administered one hour prior |
| Kainate-induced SE in mice | Aqueous extract of *Syzygium cumini* | (Kandeda et al., 2022) | Extract prevented SE and mortality, improved memory, reduced oxidative stress and neuroinflammation in the hippocampus. | Antiepileptic and anti-amnesic effects mediated by antioxidant and anti-inflammatory activities. | 28.8, 72, 144, and 288 mg/kg administered orally (po). Given one hour prior |
| PTZ-induced seizures in rats | Ascorbate (Vitamin C) | (Schneider Oliveira et al., 2004) | High doses of ascorbate (300 mg/kg) protected against PTZ-induced convulsions, protein carbonylation, and Na⁺/K⁺-ATPase inhibition. Intermediate doses potentiated convulsions without affecting oxidative damage. Low doses prevented oxidative damage but not convulsions. | Anticonvulsant activity not directly related to antioxidant action; ascorbate shows a dual role depending on dose. | Administered intraperitoneally (i.p.): 30 mg/kg  100 mg/kg  300 mg/kg |
| PTZ-induced epilepsy in rats chronically treated with VPA | Astaxanthin (ASTA) | (Ata Yaseen Abdulqader et al., 2021) | ASTA exhibited anticonvulsant and anti-inflammatory effects; co-administration with VPA had synergistic effect; improved antioxidant levels, reduced TNF-α, enhanced GSH levels. | Antiepileptic and anti-inflammatory effects by reducing ROS generation; synergy with VPA in treating epilepsy. | Astaxanthin (ASTA): 100 mg/kg, Oral gavage daily.  Valproic Acid (VPA): 500 mg/kg, Oral gavage daily. |
| PTZ-induced kindling in rats | *Buxus hyrcana* extract (BHE) | (Azizi et al., 2018) | BHE prevented memory deficit caused by PTZ-induced kindling; attributed to amelioration of oxidative stress damage through antioxidant mechanism. | Antioxidant effects preventing memory deficits associated with epilepsy. | 300 mg/kg administered intraperitoneally.  600 mg/kg administered intraperitoneally. |
| PTZ-induced seizures in rats | Caffeine | (Souza et al., 2013) | Long-term caffeine supplementation decreased seizure duration, increased GSH content, protected against oxidative stress, prevented decrease in Na⁺/K⁺-ATPase activity. Effect reversed by GSH synthesis inhibitor. | Neuroprotection via enhancement of antioxidant defenses through increased GSH levels. | 5 mg/kg, 50 mg/kg,  100 mg/kg, 400 mg/kg, administered intraperitoneally (i.p.) |
| Seizures induced by pilocarpine, picrotoxin, and PTZ in mice | Carvacryl acetate (CA) | (Pires et al., 2015) | CA exhibited anticonvulsant effects, increased GABA levels, increased Na⁺/K⁺-ATPase and δ-ALA-D activities. Effects reversed by flumazenil. | Modulation of GABAergic system and enhancement of enzyme activities contributing to anticonvulsant effects. | 100 mg/kg, administered intraperitoneally (i.p.) |
| Pilocarpine-induced epilepsy in mice | *Chrysanthellum americanum* aqueous extract | (Nguezeye et al., 2023) | Extract increased latency to seizures, decreased number and duration of seizures, improved antioxidant defenses, increased acetylcholine levels. | Anticonvulsant effects mediated by enhancement of antioxidant defense system and cholinergic neurotransmission. | Administered orally 27.69 mg/kg, 69.22 mg/kg, 138.45 mg/kg, 276.90 mg/kg |
| PTZ-induced seizures in rats | Creatine | (Rambo et al., 2013) | Creatine prevented EEG changes, increased seizure latencies, reduced seizure duration, prevented decrease in ATP levels and Na⁺/K⁺-ATPase activity, prevented mitochondrial dysfunction. | Energy metabolism support and mitochondrial protection contributing to anticonvulsant effects. | Acutely at a dosage of 300 mg/kg orally (p.o.) |
| PTZ-induced kindling in rats | Curcuma zedoaria (CZ) extract | (Mahmoudi et al., 2020) | CZ extract increased tonic seizure threshold, reduced mortality, improved memory and learning, increased antioxidant capacity, decreased MDA and NO levels in brain and serum. Flumazenil reduced these effects, suggesting involvement of GABAergic system. | Antioxidant effects and modulation of GABAergic neurotransmission contributing to neuroprotection and cognitive improvement. | 100 mg/kg, 200 mg/kg, 400 mg/kg, Administered intraperitoneally (i.p.) |
| PTZ-induced epilepsy in mice | Daidzin | (Kazmi et al., 2020) | Daidzin reduced severity of epileptogenesis, prevented histopathological changes, improved antioxidant levels, decreased MDA and nitrite, modulated BDNF/VEGF and apoptotic signaling. | Neuroprotective and anti-epileptic properties through modulation of oxidative stress, BDNF/VEGF, and apoptotic pathways. | 1 mg/kg, 5 mg/kg, 10 mg/kg, Administered intraperitoneally (i.p.) |
| PTZ-induced acute seizures in rats | Echinops spinosus extract (ESE) | (Alkhudhayri et al., 2023) | ESE increased latency to seizure onset, decreased seizure duration, prevented release of pro-inflammatory cytokines (IL-1β, TNF-α, COX-2), prevented hippocampal cell death, corrected GABA imbalance, increased Na⁺/K⁺-ATPase activity. | Antioxidant, anti-inflammatory, and antiapoptotic effects; modulation of GABA levels and Na⁺/K⁺-ATPase activity. | 250 mg/kg orally for 7 consecutive day |
| PTZ-induced seizures in mice | *Epilobium hirsutum* extract | (Yunusoğlu et al., 2020) | Extract exhibited significant anticonvulsant and antioxidant activities; regulated behavior and locomotion; inhibited MDA production and increased antioxidant enzyme levels. | Antioxidant and neuroprotective effects attributed to phenolic compounds like gallic acid. | 50, 100, 150, and 200 mg/kg of the extract were administered orally |
| PTZ-induced convulsive seizures in mice | Ethanolic extract of *Amomum tsaoko* fruits (EE-ATF) | (Wang et al., 2021) | EE-ATF decreased seizure onset and duration, suppressed seizure severity and mortality, ameliorated neurotransmitter levels (GABA, glutamate, dopamine), reduced oxidative stress, suppressed pro-inflammatory cytokine expressions, and protected neuronal integrity. | Antioxidant and anti-inflammatory effects; modulation of neurotransmitters and inhibition of pro-inflammatory pathways. | 50, 75, and 100 mg/kg orally 30 min prior |
| MES and PTZ-induced seizures in rats | Ethyl acetate extract (EAE) of Urtica dioica root | (Loshali et al., 2021) | EAE showed potent antioxidant activity, effective against MES and PTZ-induced seizures. | Antioxidant effects contributing to antiepileptic activity. | 100 mg/kg and 200 mg/kg administered orally (p.o.) |
| Kainic acid-induced seizures in mice | Ganglioside GT1b, Melatonin | (Yamamoto and Mohanan, 2003) | Both ganglioside GT1b and melatonin prevented seizures and damage to brain mitochondrial DNA (mtDNA) induced by KA. Reduced lipid peroxidation and oxidative stress observed. | Antioxidant effects through scavenging reactive oxygen species, protecting mtDNA and preventing seizures. | Melatonin: 20 mg/kg administered intraperitoneally (i.p.) Ganglioside GT1b: 90 nmol/brain administered intracerebroventricularly (i.c.v.). |
| PTZ-induced epilepsy in rats | Ginkgo biloba extract (GbE) and L-carnitine (LC) | (Essawy et al., 2022) | Pre- and post-treatment with GbE and LC suppressed kindling acquisition, alleviated oxidative stress, neuromodulatory, and antiepileptic actions. | Amelioration of oxidative/antioxidative imbalance; neuromodulatory effects. | Ginkgo biloba extract (GbE): Administered orally at 100 mg/kg.  L-carnitine (LC): Administered orally via gavage at 300 mg/kg. |
| Kainic acid-induced epilepsy in mice | Hyperoside (HYP) | (Cao et al., 2020) | HYP protected against neuronal damage in the hippocampal CA3 region, enhanced antioxidant levels, reduced autophagy via PI3K/Akt and MAPK pathways. | Antioxidant and anti-autophagy effects mediated by modulation of PI3K/Akt and MAPK signaling pathways. | Pretreatment at 50 mg/kg |
| Pilocarpine-induced seizures in rats | Idebenone | (Ahmed, 2014) | Idebenone prolonged latency to seizures, reduced seizure incidence, increased survival, reduced oxidative stress markers, prevented Na⁺/K⁺-ATPase inhibition. | Antioxidant effects and protection of Na⁺/K⁺-ATPase activity contributing to neuroprotection. | 50 mg/kg, 100 mg/kg, and 200 mg/kg administered intraperitoneally (i.p.) for 3 consecutive days |
| Kainic acid and pilocarpine-induced SE in rats | Inhibition of succinate dehydrogenase (SDH), malate/aspartate shuttle (MAS), and purine nucleotide cycle (PNC) | (Zhang et al., 2020) | Reducing succinate accumulation decreased oxidative stress, mitochondrial ROS levels, neuronal degeneration, and lessened seizure severity. Simulating succinate accumulation induced seizures similar to those observed with KA. | Succinate accumulation contributes to increased oxidative stress and mitochondrial ROS, leading to neuronal damage and seizures. | Aminooxyacetate (AOA): 31.25 μg/5 μL in saline, injected into the lateral ventricles 30 minutes before KA.  Dimethylmalonate (DM): 1 mL/kg (1.15 g/mL dissolved in ethanol, then saline), injected intraperitoneally 15 minutes before KA.  Succinic Acid Dimethyl Ester (SAD): 100 μg/2 μL in DMSO, continuously injected into the lateral ventricle for 30 minutes.  5-Aminoimidazole-4-carboxamide-1-b-d-ribofuranoside (AICAR): 1.5 μg/5 μL in DMSO, injected into the lateral ventricles 60 minutes before KA. |
| FeCl3-induced epilepsy in rats | Jujuboside A | (Lu et al., 2022) | Jujuboside A improved recognition deficiency and epilepsy syndromes, relieved oxidative stress and inflammatory responses, reduced expression of p-P38 and p-ERK1/2. | Antiepileptogenic effect by alleviating oxidative stress and inflammatory responses via p38 and ERK1/2 pathways. | Administered intracerebroventricularly (ICV) 0.02 mg/kg/day for five consecutive days |
| Pilocarpine-induced SE in rats | Lacosamide | (Shishmanova-Doseva et al., 2021) | Lacosamide attenuated number of spontaneous motor seizures, corrected impaired behaviors, suppressed oxidative stress, alleviated catalase activity, lipid peroxidation, mitigated neuroinflammation, and provided neuroprotection. | Antioxidant, anti-inflammatory, and neuroprotective activities contributing to anticonvulsant effects. | Topiramate (TPM): 40 mg/kg and 80 mg/kg, administered intraperitoneally (i.p.).  Lacosamide (LCM): 10 mg/kg and 30 mg/kg, administered intraperitoneally (i.p.). |
| Pilocarpine-induced SE in mice | Lamotrigine | (Onishi et al., 2023) | Lamotrigine prolonged latency to SE, decreased mortality rate, reduced hippocampal NO and MDA levels, increased GSH levels. Antioxidant effects not fully exerted via NMDA-related pathways. | Anticonvulsant and antioxidant effects mitigating oxidative stress; mechanisms independent of NMDA receptors. | 20 mg/kg, administered intraperitoneally (i.p.) 30 minutes before pilocarpine |
| Pilocarpine-induced seizures in rats | Lipoic Acid (LA) | (de Sales Santos et al., 2010) | LA preadministration abolished seizures, reversed decreased δ-ALA-D and Na⁺/K⁺-ATPase activities caused by seizures. | Antioxidant properties increasing δ-ALA-D and Na⁺/K⁺-ATPase activities, contributing to seizure prevention. | 10 mg/kg, administered intraperitoneally (i.p.) 30 minutes before pilocarpine administration |
| PTZ-induced seizures in rats | Lycopene | (Taskiran and Tastemur, 2021) | Lycopene prolonged seizure onset, reduced seizure stages, improved memory impairment, reduced levels of iNOS, nNOS, and NO in brain regions. | Anticonvulsant effect associated with modulation of nitric oxide pathway; antioxidant properties reducing oxidative stress. | 5 and 10 mg/kg/day for ten days. |
| PTZ-induced epilepsy in rats | Melatonin | (Şirinyıldız and Ek, 2021) | Melatonin showed protective role against physiological changes caused by epilepsy, reduced oxidative damage, increased antioxidant potency, dose-dependent effects observed. | Antioxidant properties reducing oxidative stress and enhancing antioxidant defenses. | 25, 100 mg/kg: Administered intraperitoneally (i.p.) 30 minutes before each PTZ injection |
| Pilocarpine-induced epilepsy in rats | Melissa officinalis extract (MOE) | (Abd Allah et al., 2022) | MOE prevented neuronal loss, decreased oxidative stress, inflammation, enhanced GSH and antioxidant enzymes, upregulated Nrf2/HO-1 pathway, increased GABA and Na⁺/K⁺-ATPase activity, decreased glutamate and acetylcholine levels. | Antioxidant and anti-inflammatory effects via activation of Nrf2/HO-1 pathway; modulation of neurotransmitters and Na⁺/K⁺-ATPase activity. | 250 mg/kg: Administered orally daily for 2 weeks |
| PTZ-kindled epilepsy in rats | Methanolic extract of Biarum carduchrum | (Amini et al., 2020) | Extract reduced seizures, improved memory and learning, reduced nitric oxide and malondialdehyde levels, increased antioxidant capacity. | Antiepileptic effects associated with antioxidant properties and modulation of oxidative stress. | 100, 200 mg/kg: Administered intraperitoneally (i.p.) daily for 10 days |
| PTZ-induced epilepsy in mice | Methanolic extract of Otostegia limbata | (Amin et al., 2022) | Extract decreased seizure duration, increased latency, downregulated TNF-α and p-NF-κB expression, reduced oxidative stress, protected neurons. | Antioxidant and anti-inflammatory effects contributing to anticonvulsant activity. | 100, 200, 300 mg/kg: Administered orally 30 minutes before PTZ injection |
| Glutaric acid-induced seizures in rats | Monosialoganglioside (GM1) | (Fighera et al., 2006) | GM1 protected against GA-induced seizures, oxidative damage, and Na⁺/K⁺-ATPase inhibition. Muscimol also provided protection; NMDA receptor antagonists did not. | Protection of Na⁺/K⁺-ATPase activity and modulation of GABA_A receptors contributing to neuroprotection. | 50 mg/kg, administered intraperitoneally (i.p.) twice |
| PTZ-induced tonic-clonic convulsions in mice | Morin | (Kandhare et al., 2018) | Morin (20 and 40 mg/kg) inhibited PTZ-induced increase in duration and onset of convulsions, decreased mortality. Restored brain GABA, dopamine, Na⁺/K⁺-ATPase levels; reduced oxidative stress markers (MDA, NO); increased SOD and glutathione levels. | Antioxidant effects and modulation of neurotransmitters contributing to anticonvulsant activity. | 10 mg/kg, 20 mg/kg, and 40 mg/kg administered intraperitoneally (i.p.) |
| In vitro seizure-like activity; status epilepticus in animals | NADPH oxidase inhibitor and Nrf2 activator (combination therapy) | (Shekh-Ahmad et al., 2019) | Combination therapy prevented excessive ROS accumulation, mitochondrial depolarization, neuronal death during in vitro seizure activity, and reduced development of spontaneous seizures after SE. | Inhibition of ROS generation and activation of endogenous antioxidant defenses via Nrf2 pathway. | AEBSF: 50 mg/kg, administered acutely  RTA 408: 25 mg/kg |
| PTZ-induced seizures in mice | Nux vomica homeopathic medicinal preparations (HMPs) | (Mishra et al., 2021) | HMPs delayed latency and reduced duration of seizures; attenuated behavioral impairment; reduced oxidative stress markers; enhanced GSH and SOD levels. | Anticonvulsant and antiepileptogenic effects through modulation of GABAergic neurotransmission and antioxidant mechanisms. | 50 µL, administered orally |
| Kainic acid-induced epilepsy in rats | Olive Leaf Extract (OLE) | (Khamse et al., 2021) | OLE significantly reduced seizure scores, decreased oxidative stress markers (MDA, nitrite, nitrate), increased glutathione (GSH) levels, exhibited anti-apoptotic effects on neurons. | Antioxidant and anti-apoptotic properties mitigating oxidative stress and neuronal apoptosis. | 300 mg/kg/day, Orally (gavage), Daily for 4 weeks |
| Pilocarpine-induced seizures in mice | Passiflora caerulea aqueous fruit extract (PCAE) | (Smilin Bell Aseervatham et al., 2020) | PCAE delayed onset of convulsions, decreased duration of clonic convulsions, improved cognitive function, reduced oxidative damage, activated cholinergic neurotransmission. | Antioxidant activity reducing oxidative stress; modulation of cholinergic system contributing to anticonvulsant effects. | 100, 200 mg/kg |
| Pilocarpine-induced seizure in mice | Pioglitazone | (Rostamian et al., 2021) | Pioglitazone increased latency to seizure onset, decreased MDA levels, elevated CAT, SOD, and GR enzymes in hippocampus. | Antioxidant effects reducing neuronal damage caused by seizures; PPAR-γ agonist activity. | 80 mg/kg, PO, 4 hours before Pilocarpine |
| Pentylenetetrazole (PTZ)-induced epilepsy in mice | Proanthocyanidins (PACs) | (Alyami et al., 2022) | PACs reduced seizure severity, oxidative stress, neuroinflammation, and neuronal apoptosis; upregulated Nrf2 expression; improved antioxidant enzyme activities. | Activation of the Nrf2 pathway leading to antioxidant effects, reducing oxidative stress, inflammation, and apoptosis. | Administered orally (p.o.) at a dose of 200 mg/kg daily for 10 days. |
| PTZ-induced seizures in mice | Quercetin | (Tavakoli et al., 2023) | Quercetin increased seizure threshold, increased TAC, decreased MDA levels, decreased gene expression of TNF-α, NLRP3, IL-1β, and iNOS in prefrontal cortex. | Anticonvulsant effect due to reduction of inflammatory responses and oxidative stress in prefrontal cortex. | 10 mg/kg, 20 mg/kg, and 40 mg/kg (i.p.) |
| Lithium-pilocarpine-induced SE in rats | Rosiglitazone | (Peng et al., 2021) | Rosiglitazone improved neuronal survival, enhanced antioxidative activity, increased Nrf2 expression, attenuated oxidative-stress-induced autophagy. Nrf2 knockdown blocked these effects. | Attenuation of oxidative stress and autophagy via upregulation of Nrf2 pathway. | Administered 1 hour before, exact dosage not explicitly stated |
| Rat model of kainic acid-induced temporal lobe epilepsy (TLE) | Royal Jelly (RJ) | (Hashemi and Ahmadi, 2023) | RJ pretreatment significantly reduced seizure scores, delayed seizure onset, decreased oxidative stress markers (MDA, TOS), increased total antioxidant capacity (TAC), and prevented neuronal damage in the hippocampus. | Antioxidant properties of RJ reduced oxidative stress and neuronal damage. | 150 mg/kg/day for 14 days |
| Intrahippocampal kainate-induced TLE in rats | Sinomenine | (Ramazi et al., 2020) | Sinomenine decreased seizure severity, incidence of SE, prevented neuronal loss, restored oxidative stress markers, partially counteracted increase of inflammatory markers. | Anticonvulsant and neuroprotective effects by reducing oxidative stress, inflammation, pyroptosis, and apoptosis. | 30 mg/kg, 50 mg/kg, Oral Gavage, Daily administration four days before |
| PTZ-induced seizures in mice | Soxhlet and macerated extracts of Pinus eldarica | (Mansouri et al., 2021) | Extracts delayed onset of seizures, reduced neuronal death, improved oxidative stress markers (decreased MDA, increased thiol levels, SOD, and CAT activities). | Neuroprotection via antioxidant properties reducing oxidative stress. | Soxhlet (Sox) Extract, Low Dose: 100 mg/kg  High Dose: 200 mg/kg  Macerated (Mac) Extract, Low Dose: 100 mg/kg  High Dose: 200 mg/kg, (i.p.) daily for 7 days. |
| PTZ-induced convulsions and kindling in mice | Standardized extract of *Phyllanthus amarus* (PA) containing phyllanthin | (Tao et al., 2020) | PA (100 and 200 mg/kg) reduced seizure duration and onset, decreased mortality, attenuated kindling. Restored brain GABA, dopamine, glutamate levels, Na⁺/K⁺-ATPase, Ca²⁺-ATPase activities, reduced oxidative stress, downregulated inflammatory markers (NF-κB, TNF-α, IL-1β, COX-2, TLR-4). | Antiepileptic effects via balancing neurotransmitters, modulating ion channels, inhibiting inflammation through NF-κB/TLR-4 pathway. | 50 mg/kg, 100 mg/kg, and 200 mg/kg via intraperitoneal injection daily for convulsion induction and on alternate days for a total duration of 15 days during the kindling phase |
| PTZ-induced epilepsy in rats | *Stevia rebaudiana* extract | (El Nashar et al., 2022) | Stevia significantly attenuated PTZ-induced seizures, improved oxidative stress markers, downregulated GFAP, IL-6, NF-κB, caspase-3, and p53, upregulated Sirt-1 and Bcl-2 in hippocampus. | Antioxidant, anti-apoptotic, and anti-inflammatory effects; involvement of Sirt-1 pathway. | 200 mg/kg, Oral (via gastric gavage), Daily for a total duration of 4 weeks |
| Kainic acid-induced status epilepticus (SE) in rats | Sulforaphane (SFN) | (Sandouka and Shekh-Ahmad, 2021) | SFN decreased ROS production, restored glutathione levels, reduced neuronal death, increased expression of Nrf2 and related antioxidant genes, improved oxidative stress markers, and increased total antioxidant capacity. | Activation of the Nrf2 pathway enhancing endogenous antioxidant defenses and neuroprotection. | 5 mg/kg/day for 5 days |
| Zebrafish model of epilepsy | *Tilia viridis* aqueous extract (TE) | (Saint Martin et al., 2024) | TE exhibited anticonvulsant activity at low concentrations, demonstrated antioxidant effects, attributed to presence of polyphenols, particularly flavonoids. | Antioxidant properties of polyphenols contributing to anticonvulsant effects. | 0.1, 1, 10, 50, 100, 250, 500 µg/mL |
| PTZ-induced seizures in mice | Triterpene 3β,6β,16β-trihydroxylup-20(29)-ene (TTHL) | (Della-Pace et al., 2013) | TTHL increased latency to seizures, reduced seizure duration, prevented oxidative damage, maintained Na⁺/K⁺-ATPase activity. Protection not due to direct antioxidant or GABAergic activity. | Protection of Na⁺/K⁺-ATPase activity contributing to anticonvulsant effects. | 30 mg/kg; i.g. |
| Maximal electroshock (MES), PTZ, and isoniazid (INH)-induced seizures in mice | *Vateria indica* bark extract (VIE) | (Alshabi et al., 2022) | VIE reduced seizure duration and severity, delayed seizure onset, increased brain GABA levels, exhibited significant antioxidant activity, and reduced mortality rates. | Positive modulation of GABAergic neurotransmission and enhancement of antioxidant capacities. | 250 mg/kg p.o. (Low Dose) and 500 mg/kg p.o. (High Dose) |
| Lithium–pilocarpine model of SE in rats | Vitamin C, Vitamin E | (Fuchs et al., 2023) | Preventive treatment with vitamin C or E ameliorated seizure-induced oxidative damage; decreased isoprostane formation, improved GSH/GSSG ratio. | Antioxidant properties reducing oxidative stress during seizures. | (i.p.)  Vitamin C (Ascorbic Acid): 250 mg/kg  Vitamin E (α-Tocopherol): 100 mg/kg  Coenzyme Q10 (Ubiquinone): 100 mg/kg  N-tert-Butyl-α-Phenylnitrone (PBN): 100 mg/kg  Ebselen: 10 mg/kg  Resveratrol: 50 mg/kg  (Oral Gavage)  Vitamin C (Ascorbic Acid): 500 mg/kg  Vitamin E (α-Tocopherol): 200 mg/kg  Coenzyme Q10 (Ubiquinone): 200 mg/kg  N-tert-Butyl-α-Phenylnitrone (PBN): 200 mg/kg  Ebselen: 20 mg/kg  Resveratrol: 100 mg/kg |
| Lithium-pilocarpine model of temporal lobe epilepsy in rats | Vitamin C, Vitamin E, S-adenosylmethionine (antioxidant cocktail) | (Schwarz et al., 2022;Xhuti et al., 2023) | Optimal reference genes for RT-qPCR identified; antioxidant cocktail with IL-1ra affected gene expression; Actb and B2m genes increased after SE, unsuitable as reference genes. | Combined antioxidant and anti-inflammatory effects during epileptogenesis; importance of accurate gene expression normalization in studies. | Vitamin C at 100 mg/kg, vitamin E at 200 mg/kg, and S-adenosylmethionine at 25 mg/kg intraperitoneally daily for seven days |
| PTZ-kindled epileptic rat model | Vitamin E | (Zhang et al., 2022) | Vitamin E decreased seizure severity, reduced ferroptosis markers (15-LOX expression, MDA, iron accumulation), increased GPX4 and GSH expression, and protected hippocampal neurons. | Inhibition of ferroptosis by reducing 15-LOX expression; antioxidant effects preserving neuronal integrity. | 200 mg/kg intraperitoneally 15 times every other day for 29 days |
| PTZ-induced epilepsy in rats | Zinc sulfate | (Mehmet et al., 2022) | Zinc decreased spike percentages and seizure severity, reduced MDA levels, increased SOD and HSP-70 levels. | Antiepileptogenic effects by alleviating oxidative stress and neuroinflammation. | 50, 100 mg/kg i.p, |

Table 1: Various studies that have investigated the effects of different antioxidants in animal models of epilepsy. Abbreviations: AA (Ascorbic Acid); AATP (Ascorbic Acid, Alpha-Tocopherol, Sodium Pyruvate); AChE (Acetylcholinesterase); AED (Antiepileptic Drug); AMPA (α-Amino-3-hydroxy-5-methyl-4-isoxazolepropionic Acid); AMPK (AMP-Activated Protein Kinase); ASTA (Astaxanthin); BChE (Butyrylcholinesterase); BDNF (Brain-Derived Neurotrophic Factor); BHE (*Buxus hyrcana* Extract); CA (Carvacryl Acetate); CAT (Catalase); COX-2 (Cyclooxygenase-2); Cr (Creatine); CZ (*Curcuma zedoaria*); DCFH-DA (Dichlorofluorescein Diacetate); DTDS (2,2'-Dithienyl Diselenide); EAE (Ethyl Acetate Extract); EEG (Electroencephalogram); EPM (Elevated Plus Maze Test); ESE (*Echinops spinosus* Extract); GA (Glutaric Acid); GABA (Gamma-Aminobutyric Acid); GbE (*Ginkgo biloba* Extract); GFAP (Glial Fibrillary Acidic Protein); GSH (Reduced Glutathione); GSSG (Oxidized Glutathione); GSH/GSSG (Ratio of Reduced to Oxidized Glutathione); HIF1α (Hypoxia-Inducible Factor 1-Alpha); HMP (Homeopathic Medicinal Preparation); HO-1 (Heme Oxygenase-1); IL-1β (Interleukin-1 Beta); INH (Isoniazid); iNOS (Inducible Nitric Oxide Synthase); KA (Kainic Acid); LCM (Lacosamide); MDA (Malondialdehyde); MOE (*Melissa officinalis* Extract); mtDNA (Mitochondrial DNA); NADPH (Nicotinamide Adenine Dinucleotide Phosphate); NF-κB (Nuclear Factor Kappa-Light-Chain-Enhancer of Activated B Cells); NMDA (N-Methyl-D-Aspartate); NO (Nitric Oxide); Nrf2 (Nuclear Factor Erythroid 2–Related Factor 2); OLE (Olive Leaf Extract); PA (*Phyllanthus amarus*); PACs (Proanthocyanidins); PCAE (*Passiflora caerulea* Aqueous Extract); PHEN (Phenobarbital); PIC (Picrotoxin); PNC (Purine Nucleotide Cycle); PTZ (Pentylenetetrazole); QTCA-1 (1-(7-Chloroquinolin-4-yl)-5-Methyl-N-Phenyl-1H-1,2,3-Triazole-4-Carboxamide); RJ (Royal Jelly); ROS (Reactive Oxygen Species); SDH (Succinate Dehydrogenase); SE (Status Epilepticus); SFN (Sulforaphane); SOD (Superoxide Dismutase); TAC (Total Antioxidant Capacity); TBARS (Thiobarbituric Acid Reactive Substances); TLE (Temporal Lobe Epilepsy); TNF-α (Tumor Necrosis Factor-Alpha); TOS (Total Oxidant Status); TPP⁺ (Triphenylphosphonium); TTHL (Triterpene 3β,6β,16β-Trihydroxylup-20(29)-ene); δ-ALA-D (Delta-Aminolevulinic Acid Dehydratase); ΔΨ (Mitochondrial Membrane Potentia
